# Predicting acute brain lesions on magnetic resonance imaging in acute carbon monoxide poisoning: a multicenter prospective observational study

**DOI:** 10.1038/s41598-023-49216-9

**Published:** 2023-12-13

**Authors:** Kyung Hun Yoo, Hyunggoo Kang, Jaehoon Oh, Tae Ho Lim, Yongil Cho, Juncheol Lee, Sang Hwan Lee, Seungkyo Jung, Won Young Kim, Chang Hwan Sohn, Byuk Sung Ko

**Affiliations:** 1 Department of Emergency Medicine, College of Medicine, Hanyang University, 222 Wangsimni-ro, Seongdong-gu, Seoul, 04763 South Korea; 2 Department of Emergency Medicine, University of Ulsan College of Medicine, Asan Medical Center, 88 Olympic-ro 43-gil, Songpa-gu, Seoul, 05505 South Korea

**Keywords:** Brain injuries, Neurotoxicity syndromes, Predictive markers, Brain imaging

## Abstract

An acute brain lesion (ABL) identified by brain magnetic resonance imaging (MRI) after acute carbon monoxide (CO) poisoning is a strong prognostic factor for the development of delayed neuropsychiatric syndrome (DNS). This study aimed to identify predictors of ABLs on MRI in patients with acute CO poisoning. This was a multicenter prospective registry-based observational study conducted at two tertiary hospitals. A total of 1,034 patients were included. Multivariable logistic regression analysis showed that loss of consciousness (LOC) (adjusted odds ratio [aOR] 2.68, 95% Confidence Interval [CI]: 1.49–5.06), Glasgow Coma Scale (GCS) score < 9 (aOR 2.41, 95% CI: 1.49–3.91), troponin-I (TnI) (aOR 1.22, 95% CI: 1.08–1.41), CO exposure duration (aOR 1.09, 95% CI: 1.05–1.13), and white blood cell (WBC) (aOR 1.05, 95% CI: 1.01–1.09) were independent predictors of ABLs on MRI. LOC, GCS score, TnI, CO exposure duration, and WBC count can be useful predictors of ABLs on MRI in patients with acute CO poisoning, helping clinicians decide the need for an MRI scan or transfer the patient to an appropriate institution for MRI or hyperbaric oxygen therapy.

## Introduction

Carbon monoxide (CO) poisoning is a leading cause of poisoning-related deaths in the United States, responsible for more than 50,000 emergency department (ED) visits and 6000 deaths annually, and possibly accounting for more than half of all fatal poisonings worldwide^1,2^. As CO inhalation has become a prevalent method of suicide in Asian nations, the incidence of CO poisoning has increased rapidly in recent years^3–5^. Despite diverse efforts including ongoing public education, enhanced effectiveness of residential CO alarms, and increasingly effective therapeutic management, the global incidence of CO poisoning has not decreased^6,7^.

CO binds to hemoglobin with an affinity 200–250 times that of oxygen, producing carboxyhemoglobin (COHb), resulting in severe tissue hypoxia^8^. In addition to hypoxia-induced tissue and cell damage, CO exposure causes lipid peroxidation, leading to progressive demyelination and inflammation in the cerebral white matter^9^. As a result, 15–40% of acute CO poisoning patients suffer from delayed neuropsychiatric syndrome (DNS)^10^. Although 50–75% of patients with DNS recover within a year, the condition can be permanent in about 25% of cases, even resulting in death despite appropriate treatment^10–12^. Therefore, preventing DNS occurrence has become a major goal of CO poisoning treatment. The search for predictive factors of DNS occurrence have yielded inconsistent results across studies, with varying degrees of accuracy^13,14^. A recent study suggested that the presence of an acute brain lesion (ABL) on magnetic resonance imaging (MRI) is strongly associated with an increased risk of DNS occurrence, with patients who exhibit ABLs on MRI having a 14-fold greater risk of DNS occurrence compared to those without ABLs^15^. Consequently, brain MRI is an important diagnostic tool for predicting DNS occurrence and assessing brain damage in patients with acute CO poisoning^16^. The identification of ABLs by MRI is crucial in predicting the patient's prognosis and determining the necessity of additional treatment, such as hyperbaric oxygen therapy (HBOT).

However, MRIs are difficult to perform on all patients with CO poisoning due to a multitude of factors, including cost, inefficient use of medical resources, lack of MRI equipment, and the unstable condition of the patient. Therefore, before performing MRI on patients with acute CO poisoning, it is important to identify the predictive factors of ABLs on MRI. In this study, we aimed to identify independent predictive factors of ABLs on MRI in patients with acute CO poisoning.

## Methods

### Study design

This multicenter registry-based observational study was performed at two tertiary academic hospitals in Seoul, Republic of Korea. The study design was ethically approved by the Institutional Review Board (IRB) of Hanyang University Hospital (HYUH 2022–07-014) and Asan Medical Center (IRB no. 2022–1118). The need for written informed consent was waived by the IRB of Hanyang University Hospital and the IRB of Asan Medical Center due to the observational nature of this study. This study was conducted in accordance with the principles outlined in the Declaration of Helsinki.

### Study population

The registry included all acute CO poisoning patients who visited the emergency departments (ED) of Hanyang University Hospital (Seoul, Korea) from January 2017 to December 2022 and Asan Medical Center (Seoul, Korea) from January 2008 to December 2015. The development of this registry was initiated with the primary objective of providing comprehensive support for research focused on the field of CO poisoning. The registry inclusion criteria included obvious evidence of CO exposure in the patient’s history or an initial arterial blood COHb level > 5% in nonsmokers and > 10% in smokers as measured at the ED^17^. Given the half-life of COHb, initial COHb levels estimated to be > 10% in smokers and > 5% in nonsmokers were also included^18^. The purpose of this study was to provide an analysis of adult patients who develop ABL on MRI as a result of CO poisoning. Therefore, patients under the age of 18 and those who did not undergo brain MRI were excluded.

### Data collection

The following clinical characteristics were collected: age, sex, vital signs, comorbidities, smoking status, exposure type (accidental or intentional), Glasgow Coma Scale (GCS) score, loss of consciousness (LOC), co-ingestion, duration of CO exposure, interval from last CO exposure to ED arrival, ABL on brain MRI, and laboratory findings (lactate levels, arterial pH, base excess, bicarbonate, COHb levels, white blood cell [WBC] count, C-reactive protein [CRP] levels, hemoglobin count, platelet count, creatinine levels, blood urea nitrogen levels, creatine kinase [CK] levels, and troponin I [TnI] levels.

### Evaluation of ABLs on brain MRI

An ABL was defined as significant high signal intensity on a diffusion-weighted image (DWI) in patients with acute CO poisoning. However, changes in high signal intensity due to the T2 shine-through effect of chronic lesions were not defined as ABLs^15,19^. In this study, 3-T (Ingenia Edition X, Philips Healthcare, Amsterdam, Netherlands and Achieva, Philips Healthcare, Amsterdam, Netherlands) and 1.5-T (Avanto, Siemens Healthcare, Erlangen, Germany) MRI scanners were used at the Hanyang University Hospital and Asan Medical Center, respectively. The MRI protocol consisted of apparent diffusion coefficient (ADC) map (b0 DWI, b1000 DWI, ADC) and fluid-attenuated inversion recovery (FLAIR) images. Brain MRI was recommended for all patients with CO poisoning who fulfill the inclusion criteria outlined in the registry. Brain MRI was performed between 24–48 h after initial CO exposure. All images were read by neuro-radiologic specialists.

### Management

Patients with acute CO poisoning who visited the ED through emergency medical services were administered normobaric oxygen therapy (NBOT) at a flow rate of 15 L/min via a non-rebreathing mask during transport. Once patients arrived at the ED, they were treated with NBOT, which was continued until HBOT was performed or until ED discharge. Furthermore, HBOT was administered if the following conditions were met: arterial blood tests revealed a COHb level > 25% or > 15% in pregnant women; there was temporary loss of consciousness; there were signs of acute myocardial ischemia; there was evidence of cardiovascular dysfunction/dysrhythmia; the patient had severe metabolic acidosis; and the patient had neurologic symptoms such as confusion, altered mental status, seizures, impaired cognitive function, or focal neurological deficits^20^. HBOT was administered for at least three sessions, unless the patient or guardian refused or there was a reason to stop, such as otalgia due to pressure change. First session of HBOT was administered as soon as possible following the patient's arrival at the ED. If the patient had persistent CO poisoning symptoms, newly developed brain lesions on MRI, signs of ischemia, or other neurological abnormalities, additional sessions were considered. The patients with newly diagnosed ABL on MRI are considered to be at a high risk for developing DNS, which may serve as an indication for the administration of HBOT. Nevertheless, as DNS can occur even in the absence of ABL on MRI, patients received three sessions of HBOT regardless of the presence of ABL. Furthermore, an additional two sessions of HBOT were administered to patients diagnosed with ABL. Hanyang University Hospital applied an HBOT protocol of at least 120 min, including 30 min of pressurization, 1-h maintenance, and 30 min of decompression, while Asan Medical Center applied a 90-min protocol of 30 min of pressurization, 30 min maintenance, and 30 min of decompression. For maintenance pressure, 2.5 atmosphere absolute pressure (ATA) was applied at both hospitals^21^.

### Statistical analysis

Continuous variables were presented as mean and standard deviation (SD) with normal distribution, and as median and interquartile range (IQR) with non-normal distribution. The Shapiro–Wilk test was used for the normality test. Categorical variables were expressed as frequency and percentile. We compared each variable according to the occurrence of ABLs on MRI. The χ^2^ test and Fisher's exact test were used for categorical variables, as appropriate. The *t*-test was used for continuous variables with normal distribution, and the Mann–Whitney U test was used for continuous variables with non-normal distribution. Variables with a p-value < 0.1 by univariate logistic regression were selected as candidate variables for the multivariable logistic regression model. A multivariable logistic regression analysis was conducted to identify predictors of ABL, and results are presented as the adjusted odds ratios (aOR) with 95% confidence interval (CI). An area under the curve (AUC) of the receiver operating characteristic (ROC) curve was calculated to determine regression model performance. In addition, the predictive accuracy of individual variables for an ABL on MRI was analyzed using a ROC curve. A two-tailed p-value < 0.05 was considered statistically significant. All statistical analyses were conducted using R software, version 4.2.2 (R: A Language and Environment for Statistical Computing, R Core Team, R Foundation for Statistical Computing, Vienna, Austria, 2023, http://www.R-project.org/).

## Results

**Figure 1:**
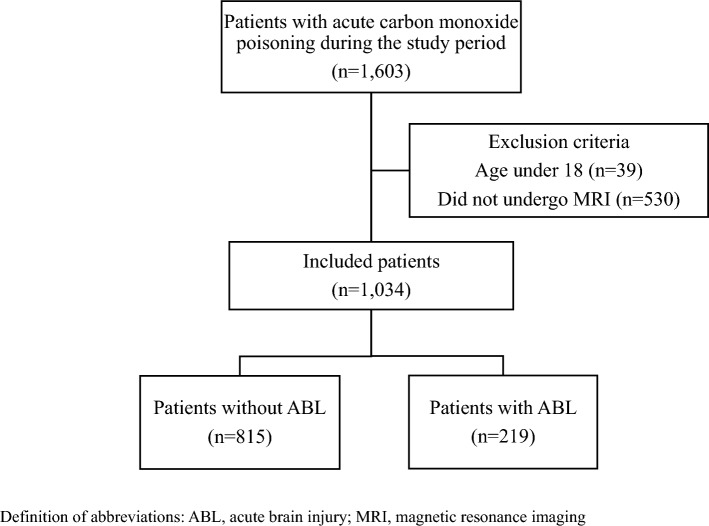
Flow diagram of the study population.

During the study period, 1603 patients with acute CO poisoning were identified (Hanyang University Hospital: 890, Asan Medical Center: 713). Of these, 569 patients were excluded for the following reasons: 39 patients were under the age of 18 (Hanyang University Hospital; 39, Asan Medical Center; 0), and 530 patients did not undergo MRI (Hanyang University Hospital: 246, Asan Medical Center: 284). Ultimately, 1034 patients were included and grouped into two: those with ABLs (219, 21%) and those without ABLs (815, 79%) (Fig. 1).

### Baseline characteristics

The baseline characteristics of the study population are summarized in Table 1. The median age of the patients was 41, and 619 were male (59.9%). Among the 1034 participants, 154 (14.9%) had hypertension, 95 (9.2%) had diabetes mellitus, 20 (1.9%) had cardiovascular disease, and 295 (28.7%) were smokers. The number of patients intentionally exposed to CO poisoning was 755 (73.0%). The median systolic blood pressure (SBP) was 124 mmHg and the median diastolic blood pressure (DBP) was 76 mmHg, while the median heart rate was 94 beats per minute (bpm). The median GCS score was 15 at the time of ED arrival, while 190 patients (18.4%) had a GCS score < 9. LOC was experienced by 731 patients (70.7%). The median time from the last CO exposure to presentation in the ED was 2.7 h, and 966 (93.9%) patients received HBOT at least once. The median CO exposure duration time was 3.7 h. Of the total study population, 127 patients (12.3%) required endotracheal intubation, and 17 patients (2.2%) required vasopressors.

**Table 1:** Baseline characteristics of the study population.

	Total patients
	(n = 1,034)
Age, years (median, IQR)	41 (31–53)
Sex, n (%)	
Male	619 (59.9)
Female	415 (40.1)
Comorbidities, n (%)	
Hypertension	154 (14.9)
Diabetes mellitus	95 (9.2)
Cardiac disease	20 (1.9)
Current smoker, n (%)	295 (28.7)
Co-ingestion	
Alcohol	378 (36.6)
Drug	239 (23.1)
Exposure type, n (%)	
Accidental	279 (27.0)
Intentional	755 (73.0)
Vital signs (median, IQR)	
Systolic blood pressure, mmHg	124 (110–138)
Diastolic blood pressure, mmHg	76 (66–86)
Heart rate, /min	94 (80–107)
Respiratory rate, /min	20 (18–22)
GCS score at presentation in the ED (median, IQR)	15 (14–15)
GCS score < 9 at presentation in the ED, n (%)	190 (18.4)
Loss of consciousness, n (%)	731 (70.7)
COHb at presentation in the ED, % (median, IQR)	16.2 (5.8–31.4)
Laboratory findings at presentation in the ED (median, IQR)	
White blood cell, × 10^3^/mm^3^	11.1 (8.0–14.9)
Platelet, × 10^3^/mm^3^	240 (206–285)
Creatinine, mg/dL	0.8 (0.6–1.0)
Creatine kinase, U/L	142 (82–443)
C-reactive protein, mg/dL	0.3 (0.1–0.5)
Troponin-I, ng/mL	0.0 (0.0–0.2)
Arterial pH	7.4 (7.4–7.4)
Bicarbonate, mmol/L	23.7 (20.5–25.6)
Lactate, mmol/L	1.9 (1.0–3.6)
CO Exposure duration, hour (median, IQR)	3.7 (1.3–8.0)
Interval from last CO exposure to presentation in the ED, hour (median, IQR)	2.7 (1.3–4.6)
Hyperbaric oxygen therapy done, n (%)	966 (93.9)
Endotracheal intubation, n (%)	127 (12.3)
Use of vasopressor, n (%)	17 (2.2)

Values are expressed as the median (interquartile range) or number (proportion). COHb, carboxyhemoglobin; CO, carbon monoxide; ED, emergency department; GCS, Glasgow coma scale.

### Comparison between groups with and without ABLs on MRI

Groups with and without ABLs differed significantly in median age (47 vs. 39 years; p < 0.001), hypertension (24.2 vs. 12.4%; p < 0.001), diabetes mellitus (15.5 vs. 7.5%; p < 0.001), cardiac disease (4.3 vs. 1.3%; p = 0.018), GCS score < 9 (37.2 vs. 13.4%; p < 0.001), LOC (89.5 vs. 65.6%; p < 0.001), WBC count (14.2 vs. 10.0 × 10^3^/mm^3^; p < 0.001), creatinine level (1.0 vs. 0.8 mg/dL; p < 0.001), CK level (1186.5 vs. 122.0 U/L; p < 0.001), CRP level (1.0 vs. 0.3 mg/dL; p < 0.001), TnI level (0.6 vs. 0.0 ng/dL; p < 0.001), bicarbonate (21.0 vs. 24.0 mmol/L; p < 0.001), lactate (2.6 vs. 1.8 mmol/L; p < 0.001), and duration of CO exposure (9.9 vs. 2.8 h; p < 0.001) (Table 2). The comparison between groups with and without ABLs on MRI for each institution is presented in Appendix Table 4, 5.

**Table 2:** Comparison of variables between the group with ABLs and the group without ABLs.

	Non-ABL	ABL	p-Value
	(n = 815)	(n = 219)	
Age, years (median, IQR)	39 (30–52)	47 (33–61)	< 0.001
Sex, n (%)			0.077
Male	476 (58.4)	143 (65.3)	
Female	339 (41.6)	76 (34.7)	
Comorbidities, n (%)			
Hypertension	101 (12.4)	53 (24.2)	< 0.001
Diabetes mellitus	61 (7.5)	34 (15.5)	< 0.001
Cardiac disease	11 (1.3)	9 (4.3)	0.018
Current smoker, n (%)	253 (29.0)	60 (27.6)	0.765
Co-ingestion			
Alcohol	304 (37.3)	74 (33.8)	0.380
Drug	202 (24.8)	37 (16.9)	0.018
Exposure type, n (%)			0.069
Accidental	231 (28.3)	48 (21.9)	
Intentional	584 (71.7)	199 (78.1)	
Vital signs (median, IQR)			
Systolic blood pressure, mmHg	124.0 (111.0–138.0)	123.0 (108.0–139.5)	0.505
Diastolic blood pressure, mmHg	75.0 (65.0–85.0)	80.0 (68.0–91.0)	0.007
Heart rate, /min	92.0 (78.0–105.0)	98.0 (85.5–113.0)	< 0.001
Respiratory rate, /min	20.0 (18.0–20.0)	20.0 (20.0–23.0)	< 0.001
GCS score < 9 at presentation in the ED, n (%)	109 (13.4)	81 (37.2)	< 0.001
Loss of consciousness, n (%)	535 (65.6)	196 (89.5)	< 0.001
COHb at presentation in the ED, % (median, IQR)	15.6 (5.9–31.2)	17.7 (5.4–31.5)	0.420
Laboratory findings at presentation in the ED (median, IQR)			
White blood cell, × 10^3^/mm^3^	10.0 (7.5–13.8)	14.2 (11.4–18.4)	< 0.001
Platelet, × 10^3^/mm^3^	241.0 (208.0–284.0)	236.0 (196.5–287.5)	0.285
Creatinine, mg/dL	0.8 (0.6–0.9)	1.0 (0.8–1.3)	< 0.001
Creatine kinase, U/L	122.0 (76.0–236.5)	1186.5 (201.0–5761.0)	< 0.001
C-reactive protein, mg/dL	0.3 (0.1–0.3)	1.0 (0.3–3.3)	< 0.001
Troponin-I, ng/mL	0.0 (0.0–0.1)	0.6 (0.1–2.0)	< 0.001
Arterial pH	7.4 (7.4–7.4)	7.4 (7.3–7.4)	0.174
Bicarbonate, mmol/L	24.0 (21.4–25.9)	21.0 (17.2–24.2)	< 0.001
Lactate, mmol/L	1.8 (1.0–3.3)	2.6 (1.4–5.5)	< 0.001
CO Exposure duration, hour (median, IQR)	2.8 (1.0–6.0)	9.9 (4.5–14.5)	< 0.001
Interval from last CO exposure to presentation in the ED, hour (median, IQR)	2.7 (1.3–4.6)	2.9 (1.2–5.2)	0.310
Hyperbaric oxygen therapy done, n (%)	767 (94.5)	199 (91.7)	0.179

Values are expressed as the median (interquartile range) or number (proportion). ABL, acute brain lesion; COHb, carboxyhemoglobin; CO, carbon monoxide; DNS, delayed neuropsychiatric sequelae; ED, emergency department; GCS, Glasgow coma scale; SpO2, saturation of percutaneous oxygen.

### Univariate and multivariable logistic regression analysis for ABLs on MRI in acute CO poisoning

The results of univariate logistic regression analysis are summarized in Table 3. Age, hypertension, diabetes mellitus, cardiac disease, drug co-ingestion, CO exposure duration, GCS score < 9, LOC, diastolic blood pressure, heart rate, and respiratory rate, in addition to WBC, creatinine, CK, CRP, TnI, bicarbonate, and lactate levels were subjected to multivariable logistic regression.

**Table 3:** Univariate and multivariable logistic regression analysis for predicting ABLs on MRI.

	Univariate analysis	p-value	Multivariable analysis	p-value
	Crude OR (95% CI)		Adjusted OR (95% CI)	
Age (years)	1.01 (1.01–1.03)	< 0.001	1.02 (1.00–1.03)	0.031
Comorbidities				
Hypertension	2.26 (1.55–3.27)	< 0.001	1.75 (0.96–3.16)	0.067
Diabetes mellitus	2.27 (1.44–3.54)	< 0.001	1.42 (0.73–2.72)	0.295
Cardiac disease	3.13 (1.25–7.67)	0.012	0.99 (0.24–3.78)	0.993
Drug co-ingestion	0.62 (0.41–0.90)	0.015	0.91 (0.54–1.51)	0.715
CO Exposure duration (hours)	1.14 (1.11–1.17)	< 0.001	1.09 (1.05–1.13)	< 0.001
GCS score < 9	3.83 (2.72–5.38)	< 0.001	2.41 (1.49–3.91)	< 0.001
Loss of consciousness	4.46 (2.89–7.21)	< 0.001	2.68 (1.49–5.06)	0.001
Vital signs				
Diastolic blood pressure (mmHg)	1.01 (1.00–1.02)	0.003	1.01 (0.99–1.02)	0.238
Heart rate (/min)	1.02 (1.01–1.02)	< 0.001	1.01 (0.99–1.02)	0.291
Respiratory rate (/min)	1.08 (1.05–1.12)	< 0.001	1.01 (0.96–1.06)	0.726
Laboratory findings				
White blood cell (× 10^3^/mm^3^)	1.12 (1.09–1.15)	< 0.001	1.05 (1.01–1.09)	0.017
Creatinine (mg/dL)	6.12 (4.04–9.55)	< 0.001	1.22 (0.71–2.09)	0.473
Creatine kinase (U/L)	1.00 (1.00–1.00)	< 0.001	1.00 (1.00–1.00)	0.057
C-reactive protein (mg/dL)	1.31 (1.23–1.41)	< 0.001	1.06 (0.97–1.16)	0.198
Troponin-I (ng/mL)	1.66 (1.47–1.90)	< 0.001	1.22 (1.08–1.41)	0.004
Bicarbonate (mmol/L)	0.91 (0.88–0.94)	< 0.001	1.02 (0.96–1.09)	0.501
Lactate (mmol/L)	1.14 (1.09–1.19)	< 0.001	1.08 (0.98–1.21)	0.134

Definition of abbreviations: ABL, acute brain lesion; CI, confidence interval; CO, carbon monoxide; GCS, Glasgow coma scale; MRI, magnetic resonance imaging; OR, odds ratio.

Multivariable logistic regression analysis showed that LOC (aOR 2.68, 95% CI: 1.49–5.06, p = 0.001), GCS score < 9 (aOR 2.41, 95% CI: 1.49–3.91, p < 0.001), TnI (aOR 1.22, 95% CI: 1.08–1.41, p = 0.004), CO exposure duration (aOR 1.09, 95% CI: 1.05–1.13), WBC (aOR 1.05, 95% CI: 1.01–1.09, p = 0.017), and age (aOR 1.01, 95% CI: 1.00–1.03, p = 0.031) were independently associated with the occurrence of ABLs on MRI (Table 3).

**Figure 2:**
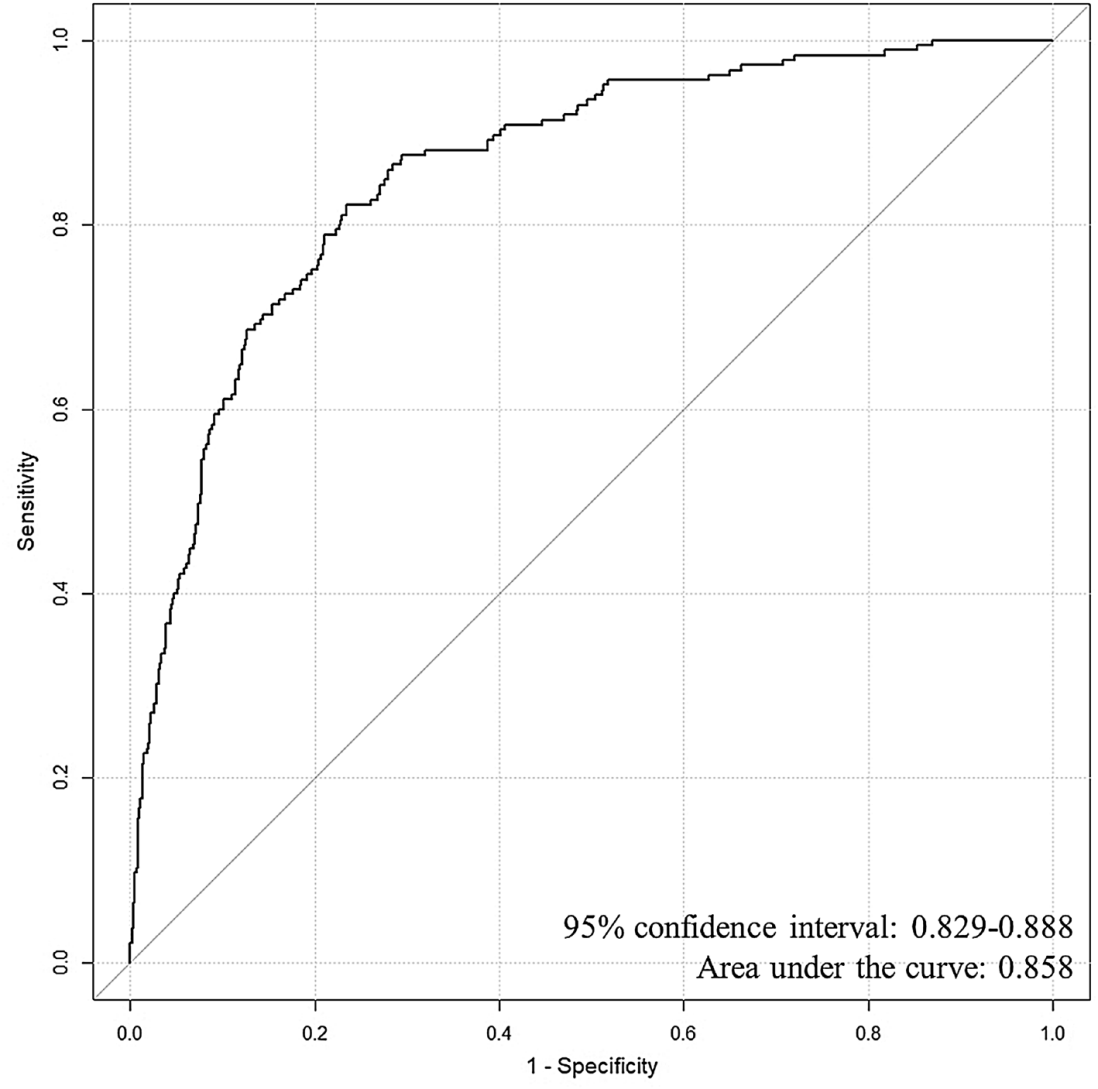
Receiver operating characteristic curve for the multiple logistic regression model.

The AUC for the multiple logistic regression model was 0.858 (95% CI: 0.83–0.89) (Fig. 2). A ROC curve analysis of TnI to determine the predictive accuracy of ABLs on MRI showed an AUC of 0.853 (95% CI: 0.83–0.88), and the optimal cut-off value by using Youden’s Index for TnI was determined to be 0.068 ng/mL, with a sensitivity of 83.6% and a specificity of 75.5% (Appendix Fig. 3). The AUC of CO exposure duration was 0.782 (95% CI: 0.75–0.82), and optimal cut-off value was > 5 h, with a sensitivity of 71.0% and a specificity of 71.1% (Appendix Fig. 4). The AUC of WBC was 0.718 (95% CI: 0.68–0.76), and optimal cut-off value was > 11.2 × 10^3^/mm^3^, with sensitivity of 78.1% and a specificity of 57.6% (Appendix Fig. 5).

## Discussion

In this study, 21.2% of patients with acute CO poisoning developed ABLs on MRI. High levels of TnI and WBC, present of LOC, GCS score < 9 at presentation, longer CO exposure duration, and older age were independently associated with ABLs on brain MRI.

Previous studies on the factors predicting the development of ABLs on MRI in patients with acute CO poisoning were performed in single institutions and had small sample sizes^22–24^. Our study was conducted at two institutions and included a relatively larger number of patients compared to previous studies. Although the registry was not purposely established for studying the development of ABLs on MRI, the strength of this study lies in its inclusion of prospective registry data with sufficient information on the general characteristics and prognostic variables of patients with acute CO poisoning. Moreover, we utilized data that had been minimized for missing values (Appendix Table 6). In the HBOT protocol, Asan Medical Center had a 30-min maintenance time and Hanyang University Hospital had a 1-h maintenance time, but other variables in the registry remained the same, indicating low heterogeneity. The schedule of MRI scans, which could potentially affect the diagnosis of ABLs on MRI, exhibited similar practice in both hospitals, with scans performed within the period of 24 to 48 h after initial CO exposure.

Reactive oxygen species produced as a result of acute CO poisoning have been found to cause damage to the endothelium of the coronary artery by affecting platelet aggregation^25,26^. Additionally, hypoxia caused by COHb impairs myocardial cell respiration, leading to direct damage to cardiac muscle^27^. TnI levels in the blood are used as an indicator of myocardial damage; an increase in TnI levels in patients with acute CO poisoning is associated with an increased risk of DNS^25^. Kim et al. reported that the initial TnI level at ED (aOR 13.66, 95% CI: 1.42–141.83) was an independent predictor for ABLs. The AUC for initial TnI at ED was 0.761 (95% CI: 0.64–0.88) and the optimal cut-off value was 0.105 ng/mL, with a sensitivity of 70.4% and a specificity of 79.2%^23^. Similarly, in our study, TnI independently predicted the occurrence of ABLs on MRI, with an AUC of 0.853. MRI timing was relatively late in the previous study, which included patients who underwent MRI within 240 h of CO exposure with a median (IQR) time of 53.21 h (39.50–67.45), whereas our study conducted MRIs 24–48 h after CO exposure and had a larger sample size (1034 patients versus 103 patients). Hence, it is probable that the differences in methodology contributed to some discrepancy in the results.

Kim et al. reported that CO exposure duration > 5 h was a predictor of ABLs in CO poisoning patients with altered mental status (aOR 7.08, 95% CI: 3.46–15.51), with an AUC of 0.815, and sensitivity and specificity of 79.1% and 69.9%, respectively^24^. In line with previous research, our study showed that a longer duration of CO exposure was found to be an independent predictor of ABLs on MRI among patients exposed to CO poisoning. Notably, our results align with previous study, further supporting the established threshold of more than 5 h as the optimal cut-off point^24^. Moreover, the findings indicated that exposure to CO for more than 5 h posed a risk for occurrence of ABLs in all patients affected by CO poisoning, regardless of their mental state.

CO poisoning leads to COHb-induced hypoxia, dysfunction of mitochondria, and increased platelet activation. Activated platelets can stimulate neutrophils to degranulate and release myeloperoxidase, thereby exacerbating the inflammatory cascade^13,28^. An increase in WBC count is a cardinal sign of the presence of acute inflammation^29^. The experimental study showed that CO-mediated DNS is linked to an adaptive immunological response^30^. Pepe et al. reported that leukocytosis (aOR 3.31; CI 95%: 1.02–10.71) is independent prognostic factor in CO poisoning patients^31^. A recent study showed that abnormal WBC count (aOR 2.57, 95% CI: 1.19–5.70) is predictor of ABL in CO poisoning patients with an altered mental status^24^. Similarly, results of this study demonstrated that an elevated WBC count is predictor of ABL in CO poisoning patients.

Previous studies have suggested an association between a GCS score < 9 and the development of DNS and/or ABLs^22,23,32^. Results from our study were comparable. A GCS score < 9 is clinically useful not only as a predictor of DNS, but also as a predictor of ABLs. LOC has been reported as a predictive factor for DNS, but not as an independent predictor of ABLs^22–24,33^. However, our study showed that LOC was a statistically significant independent predictor of ABL. O'Donnell et al. evaluated 19 patients who had suffered from CO poisoning and subsequently experienced LOC and found that 68% (13 out of 19) showed abnormalities in their brain MRI scans^34^. Among the 13 patients who exhibited abnormalities on brain MRI, it was observed that 23% (3 out of 13) had a GCS score > 9 ^34^. Changes in the mental status of patients with CO poisoning, which is often transient in nature, suggests that the presence of a LOC can serve as a useful clinical indicator for predicting the development of both DNS and ABL.

HBOT rapidly eliminates CO from circulatory system and results in beneficial outcomes on CO-induced brain injury. These benefits include reducing lipid peroxidation and decreasing the migration of endothelial leukocytes^35^. Consequently, HBOT is thought to be beneficial for preventing DNS in patients with CO poisoning which is suggested to be an important part of early intervention^33,35,36^. However, the optimal number of HBOT sessions for patients with CO poisoning remains controversial^37^. A previous nationwide cohort study reported that patients who received two or more sessions of HBOT showed better clinical outcomes in comparison to those who received a singular session of therapy^38^. Moreover, a case report has been showed that CO poisoning patient who recovered from the paroxysmal sympathetic hyperactivity after receiving repetitive HBOT^39^. Therefore, the administration of multiple sessions of HBOT may be helpful for patients with severe carbon monoxide poisoning. The presence of ABL is a strong prognostic indicator for patients with CO poisoning, and predicting its occurrence can help decisions regarding therapeutic interventions^15^. Unfortunately, our data did not include information on the occurrence of DNS, so we could not analyze the association between HBOT and the occurrence of DNS.

This study had several limitations. First, although the study was conducted using prospectively collected data from two tertiary hospitals, it is difficult to generalize the results of our study to all CO poisoning patients. Second, since not all CO poisoning patients underwent a brain MRI, there is a potential risk of selection bias. It is difficult to establish that the findings of this study adequately reflected the general characteristics of all acute CO poisoning patients because approximately one-third (530 out of 1603) of the enrolled patients were excluded because brain MRI was not performed. The recommendation for a brain MRI scan among patients enrolled in registry was not conducted selectively. Instead, all patients were recommended to receive an MRI. The majority of patients who declined the MRI scan cited financial concerns. Third, the possible effects of co-ingestion of alcohol or drugs were not considered. In the case of CO poisoning as a suicide method, patients often consume alcohol or drugs^40^, which may concurrently have an effect on the patient's altered state of consciousness. Due to the effect of co-ingested alcohol or drugs, the initial GCS score upon presentation to the ED may have been overestimated. Nevertheless, the levels of TnI and WBC are unaffected by alcohol and drug use and can be used as predictors of ABLs on MRI.

In summary, it may be useful to predict the occurrence of ABLs on brain MRI using TnI, GCS score, LOC, CO exposure duration, and WBC count in patients with acute CO poisoning. Moreover, it may help clinicians to decide if a patient should be transferred to an institution with MRI or HBOT capability. However, patients who did not undergo MRI were excluded from the analysis. Therefore, well-designed, large-scale studies are needed for more conclusive results.

## Figures and Tables

**Figure 3:**
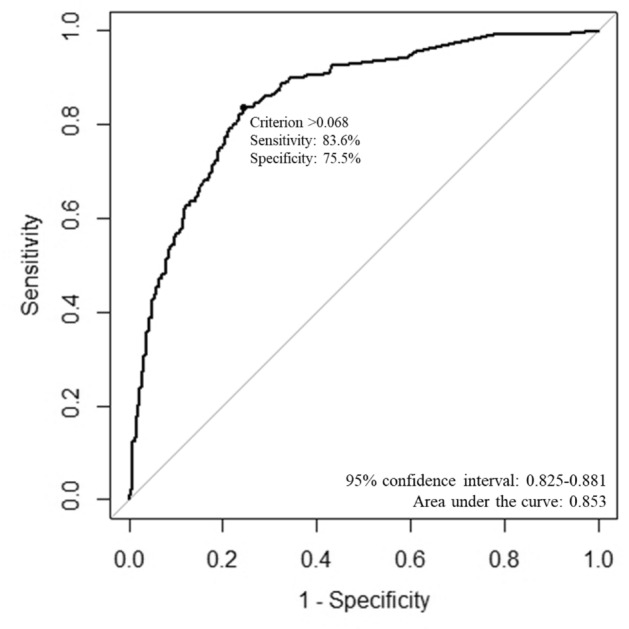
The receiver operating characteristic curve for troponin I.

**Figure 4:**
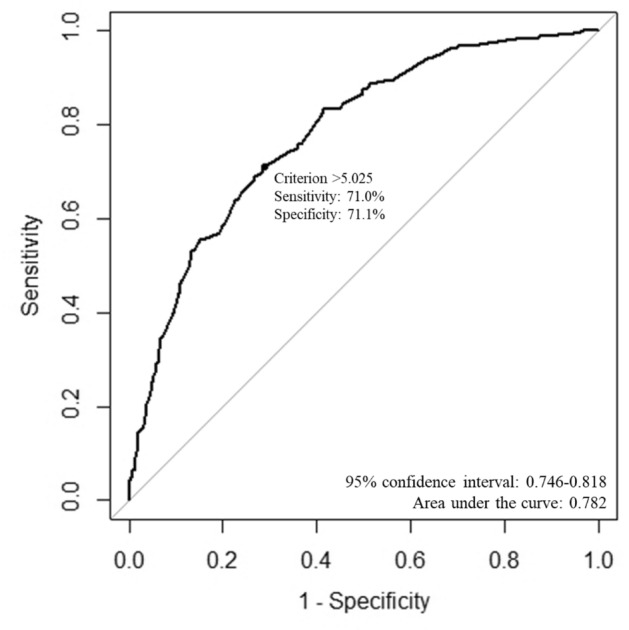
The receiver operating characteristic curve for carbon monoxide exposure duration.

**Figure 5:**
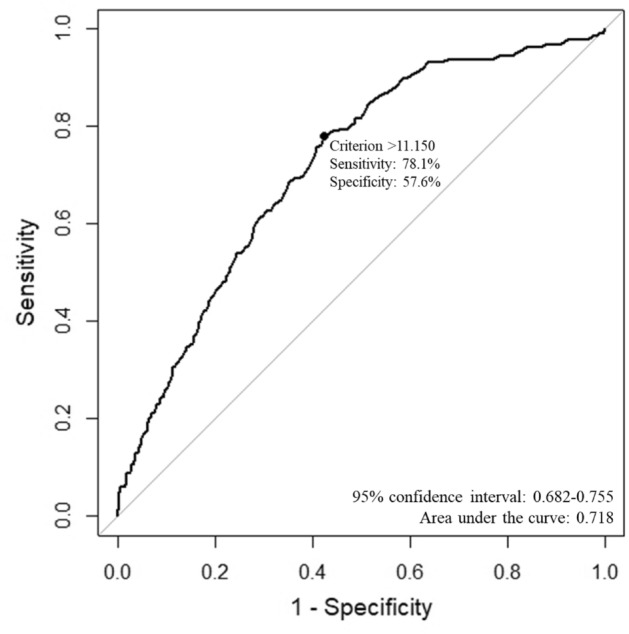
The receiver operating characteristic curve for white blood cell.

## Data Availability

The datasets used and/or analysed during the current study are available from the corresponding authors on reasonable request.

## Appendix

See Figs. 3, 4, 5 and Tables 4, 5, 6.

**Table 4:** Comparison of variables between the group with ABLs and the group without ABLs in Hanyang University Hospital.

	Non-ABL	ABL	p-value
	(n = 499)	(n = 106)	
Age, years (median, IQR)	38 (28–51)	46 (33–58)	0.005
Sex, n (%)			0.440
Male	221 (44.3)	42 (39.6)	
Female	278 (55.7)	64 (60.4)	
Comorbidities, n (%)			
Hypertension	59 (11.8)	21 (19.8)	0.041
Diabetes mellitus	36 (7.2)	14 (13.2)	0.066
Cardiac disease	6 (1.2)	6 (5.7)	0.009
Current smoker, n (%)	228 (46.1)	50 (48.1)	0.790
Co-ingestion			
Alcohol	147 (29.5)	19 (17.9)	0.022
Drug	141 (28.3)	23 (21.7)	0.208
Exposure type, n (%)			0.017
Accidental	155 (31.1)	20 (18.9)	
Intentional	344 (68.9)	86 (81.1)	
Vital signs (median, IQR)			
Systolic blood pressure, mmHg	125 (112–138)	122 (108–145)	0.578
Diastolic blood pressure, mmHg	75 (65–84)	77 (68–89)	0.113
Heart rate, /min	87 (76–100)	98 (84–111)	< 0.001
Respiratory rate, /min	20 (18–20)	20 (20–24)	< 0.001
GCS score < 9 at presentation in the ED, n (%)	26 (5.2)	28 (26.7)	< 0.001
Loss of consciousness, n (%)	272 (54.5)	84 (79.2)	< 0.001
COHb at presentation in the ED, % (median, IQR)	9.0 (3.7–17.6)	7.5 (2.6–21.5)	0.664
Laboratory findings at presentation in the ED (median, IQR)			
White blood cell, × 10^3^/mm^3^	9.7 (7.4–13.0)	13.5 (11.1–18.2)	< 0.001
Platelet, × 10^3^/mm^3^	240 (206–285)	233 (183–297)	0.557
Creatinine, mg/dL	0.8 (0.6–0.9)	1.0 (0.7–1.3)	< 0.001
Creatine kinase, U/L	119 (75–234)	1550 (203–6400)	< 0.001
C-reactive protein, mg/dL	0.3 (0.3–0.4)	1.3 (0.3–4.6)	< 0.001
Troponin-I, ng/mL	0.0 (0.0–0.0)	0.4 (0.1–1.9)	< 0.001
Arterial pH	7.4 (7.4–7.4)	7.4 (7.3–7.4)	0.051
Bicarbonate, mmol/L	23.9 (21.7–25.6)	21.2 (18.0–24.7)	< 0.001
Lactate, mmol/L	1.7 (0.9–2.9)	2.5 (1.2–5.5)	< 0.001
CO Exposure duration, hour (median, IQR)	3.0 (1.0–6.0)	9.8 (4.0–17.0)	< 0.001
Interval from last CO exposure to presentation in the ED, hour (median, IQR)	2.7 (1.1–4.7)	2.4 (1.0–5.6)	0.938
Hyperbaric oxygen therapy done, n (%)	478 (96.4)	95 (91.3)	0.047

Values are expressed as the median (interquartile range) or number (proportion). ABL, acute brain lesion; COHb, carboxyhemoglobin; CO, carbon monoxide; DNS, delayed neuropsychiatric sequelae; ED, emergency department; GCS, Glasgow coma scale; SpO2, saturation of percutaneous oxygen.

**Table 5:** Comparison of variables between the group with ABLs and the group without ABLs in Asan Medical Center.

	Non-ABL	ABL	p-value
	(n = 316)	(n = 113)	
Age, years (median, IQR)	40 (32–54)	47 (35–63)	0.003
Sex, n (%)			0.204
Male	118 (37.3)	34 (30.1)	
Female	198 (62.7)	79 (69.9)	
Comorbidities, n (%)			
Hypertension	42 (13.3)	32 (28.3)	< 0.001
Diabetes mellitus	25 (7.9)	20 (17.7)	0.006
Cardiac disease	5 (1.6)	3 (2.7)	0.750
Current smoker, n (%)	7 (2.2)	10 (8.8)	0.005
Co-ingestion			
Alcohol	157 (49.7)	55 (48.7)	0.940
Drug	61 (19.3)	14 (12.4)	0.129
Exposure type, n (%)			0.978
Accidental	27 (8.5)	9 (8.0)	
Intentional	240 (75.9)	104 (92.0)	
Vital signs (median, IQR)			
Systolic blood pressure, mmHg	123 (110–137)	123 (109–138)	0.819
Diastolic blood pressure, mmHg	76 (66–86)	81 (67–93)	0.040
Heart rate, /min	99 (84–111)	99 (88–114)	0.386
Respiratory rate, /min	20 (20–22)	20 (20–22)	0.945
GCS score < 9 at presentation in the ED, n (%)	83 (26.3)	53 (46.9)	< 0.001
Loss of consciousness, n (%)	263 (83.2)	112 (99.1)	< 0.001
COHb at presentation in the ED, % (median, IQR)	33.5 (22.0–43.8)	29.4 (17.1–38.8)	0.051
Laboratory findings at presentation in the ED (median, IQR)			
White blood cell, × 10^3^/mm^3^	10.5 (7.6–15.1)	14.3 (11.8–18.5)	< 0.001
Platelet, × 10^3^/mm^3^	243 (211–283)	237 (203–282)	0.297
Creatinine, mg/dL	0.8 (0.6–0.9)	1.0 (0.8–1.3)	< 0.001
Creatine kinase, U/L	125 (77–241)	1169 (201–5543)	< 0.001
C-reactive protein, mg/dL	0.1 (0.1–0.2)	0.7 (0.2–2.6)	< 0.001
Troponin-I, ng/mL	0.0 (0.0–0.1)	0.7 (0.1–2.4)	< 0.001
Arterial pH	7.4 (7.4–7.4)	7.4 (7.3–7.4)	0.998
Bicarbonate, mmol/L	24 (21–26)	21 (17–24)	< 0.001
Lactate, mmol/L	2.0 (1.1–3.7)	2.9 (1.5–5.8)	0.001
CO Exposure duration, hour (median, IQR)	2.5 (1.1–6.0)	9.9 (5.1–13.0)	< 0.001
Interval from last CO exposure to presentation in the ED, hour (median, IQR)	2.7 (1.5–4.3)	3.1 (1.6–4.8)	0.102
Hyperbaric oxygen therapy done, n (%)	289 (91.5)	104 (92.0)	> 0.999

Values are expressed as the median (interquartile range) or number (proportion). ABL, acute brain lesion; COHb, carboxyhemoglobin; CO, carbon monoxide; DNS, delayed neuropsychiatric sequelae; ED, emergency department; GCS, Glasgow coma scale; SpO2, saturation of percutaneous oxygen.

**Table 6:** Frequency of missing values.

Variable	Number of missing values
CO exposure duration	107
GCS score	1
Heart rate	3
Respiratory rate	11
White blood cells	2
Platelets	4
C-reactive protein	5
Creatinine	2
Troponin I	3
Bicarbonate	22
Lactate	27

CO, carbon monoxide; GCS, Glasgow coma scale.
